# Supplementary material for: Amelioration of Serum Aβ Levels and Cognitive Impairment in APPPS1 Transgenic Mice Following Symbiotic Administration
Source: Nutrients. 2024 Jul 23;16(15):2381. doi: 10.3390/nu16152381 (PMC11313784; doi:10.3390/nu16152381)
Supplement: Supplementary file 1 [file nutrients-16-02381-s001.zip › nutrients-3099620-supplementary.pdf]

Supplementary Materials:

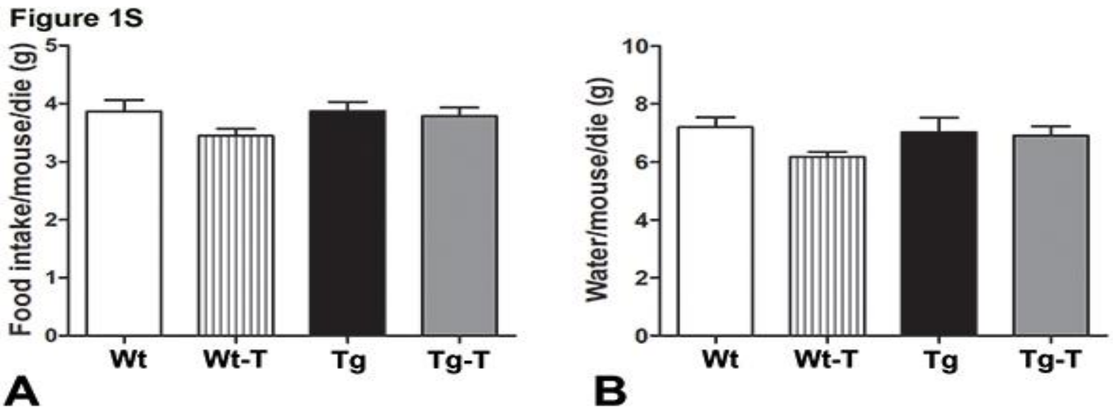

Figure S1. Food and water intake. The food and water intake were measured daily for the entire period of treatment. The results showed no differences among the groups. *One-way* ANOVA, *post-hoc* Newman-Keuls Multiple Comparison Test. Wt n= 8; Wt-T n=8; Tg n=8; Tg-T n=8.

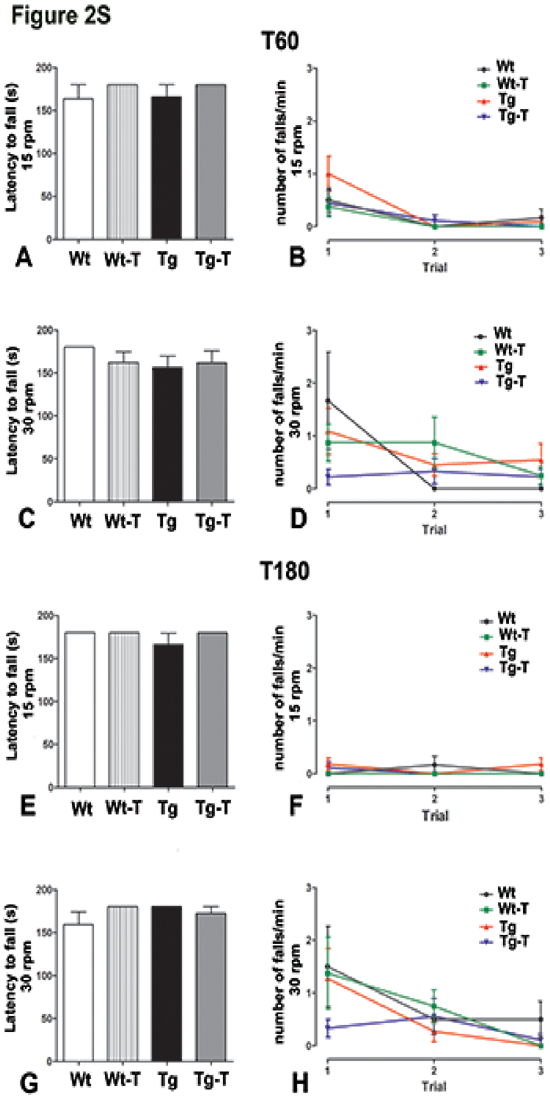

Figure S2. Rotarod test. At 4 months of life (T60) the rotarod test performed at the lowest speed (15 rpm) and at the highest speed (30 rpm) showed no significant differences between groups for both the parameters evaluated, i.e. the latency to the first fall (**A, C**) and the number of falls per min (**B, D**). At 8 months of life (T180) the parameters evaluated showed no significant differences between groups at 15 rpm speed (**E, F**) as well as at 30 rpm speed (**G, H**). *One-way* ANOVA, *post-hoc* Newman-Keuls Multiple Comparison Test. Wt n= 6; Wt-T n=8; Tg n=11; Tg-T n=9.

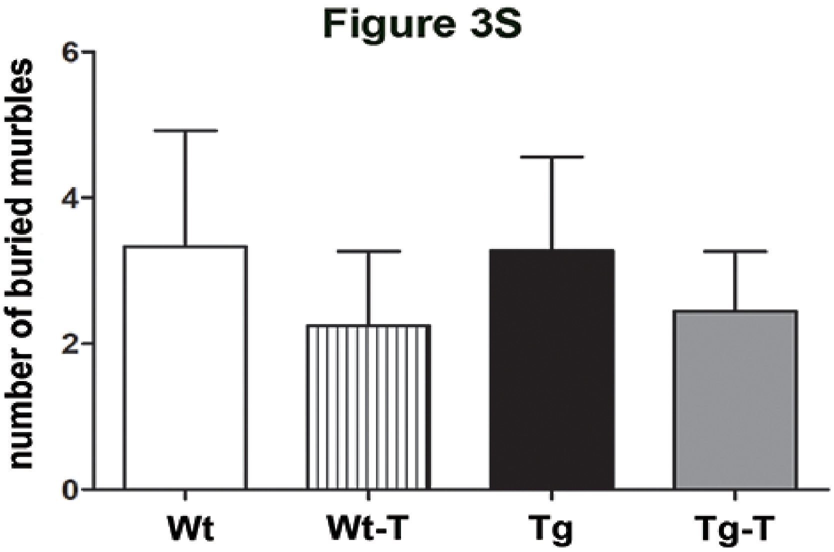

Figure S3. Marble burying test. At 8 months of life, the number of marbles buried was comparable among the experimental groups. *One-way* ANOVA, *post-hoc* Newman-Keuls Multiple Comparison Test. Wt n= 6; Wt-T n=8; Tg n=11; Tg-T n=9.

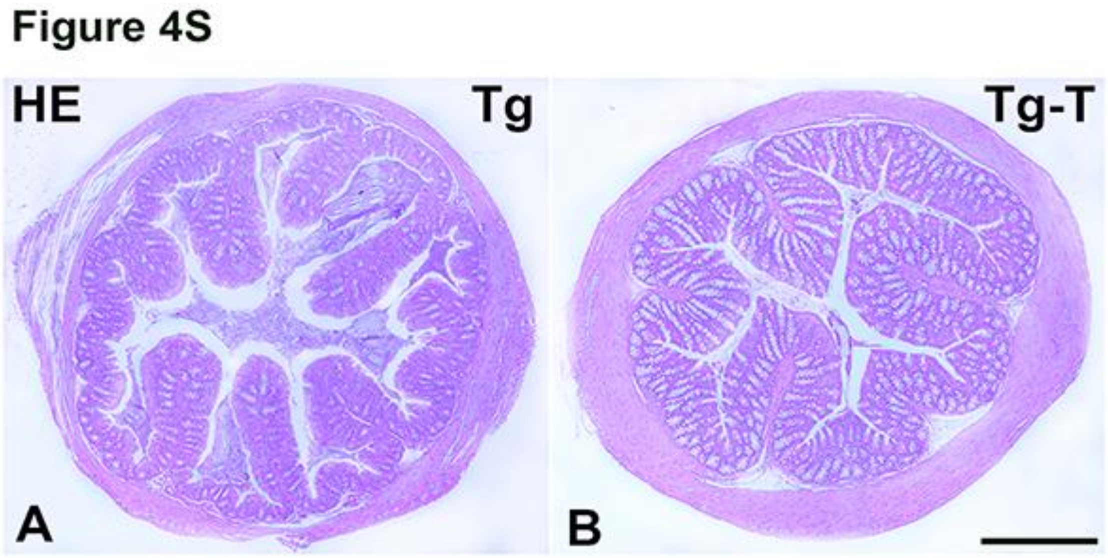

Figure S4 Hematoxylin–eosin (H&E) staining. In Tg-T mice (**B**) the mucosa shares particularly trophic and well-developed villi compared to Tg mice (**A**). Bar= 200µm.

Figure 5S

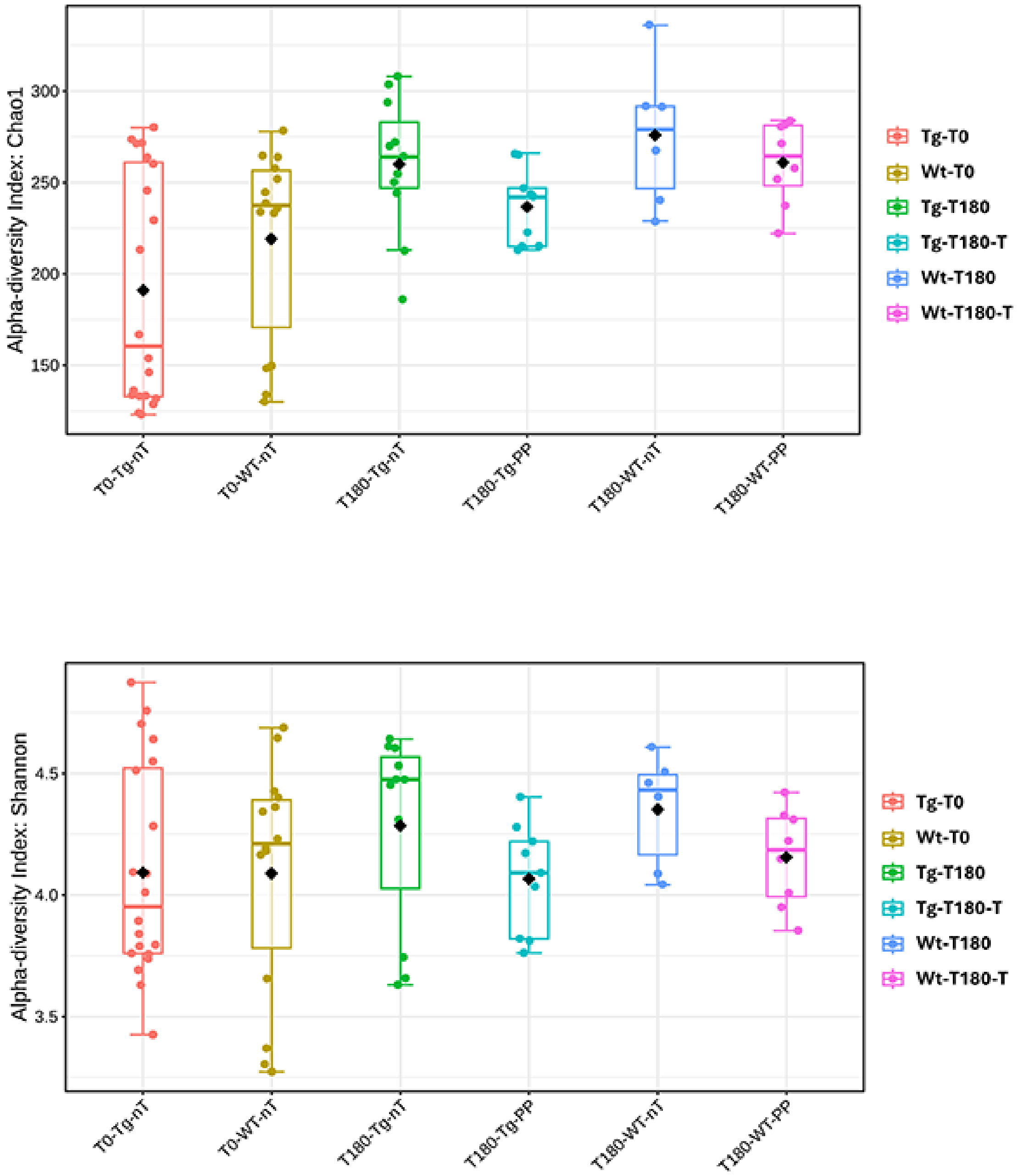

Figure S5. Analysis of α-diversity. Assessment of α-diversity using the Chao1 (upper panel) and Shannon (lower panel) diversity indexes in samples obtained from control (Ctrl) and transgenic APPPS1 (Tg) mice, before (Wt-T0, Tg-T0) and after (Wt-T180, Tg-T180) supplementation of the pre/pro-biotic (Wt-T180-T, Tg-T180-T) mixture.
